# Lessons on the non-linear path of medical progress and biological complexity from mouse models of the Brugada syndrome

**DOI:** 10.1093/europace/euae152

**Published:** 2024-06-14

**Authors:** Mathis Korseberg Stokke, Markéta Bébarová

**Affiliations:** Institute for Experimental Medical Research, Oslo University Hospital and University of Oslo, PB 4956 Nydalen, NO-0424 Oslo, Norway; Department of Physiology, Faculty of Medicine, Masaryk University, Brno, Czech Republic

**This editorial refers to ‘Genetic background determines the severity of age-dependent cardiac structural abnormalities and arrhythmia susceptibility in Scn5a-1798insD mice’, by G.A. Marchal *et al*., https://doi.org/10.1093/europace/euae153.**

Despite decades of research and early categorization as a monogenic disease, Brugada syndrome keeps on challenging clinicians and scientists alike. Key questions related to risk prediction, therapy, and individual differences in phenotype expression remain hot topics for debate.^1^ Improvements will undoubtedly depend on multicentre studies to accumulate knowledge from a sufficient number of patients suffering from this relatively rare disease with a worldwide prevalence of 0.5 per 1000,^2^ but also on improved mechanistic insight from translational studies.^3,4^ In this edition of *Europace*, Gerard A. Marchal and colleagues from the Amsterdam University Medical Center illustrate the role of translational research in this process, as well as the non-linear path of medical progress and the complexity of biology.^5^

From original descriptions of a monogenetic channelopathy with electrophysiological abnormalities, current descriptions of the Brugada syndrome comprise genetic and external modifiers resulting in an arrhythmogenic syndrome with both electrophysiological and structural features. Possible modifying genetic as well as non-genetic factors in *SCN5A*-related arrhythmias has been reviewed by Verkerk *et al*.^6^ For the Brugada syndrome and other conditions with convincingly identified pathogenic genetic variants, transgenic mouse lines play a non-replaceable role as tools in mechanistic studies due to the opportunity to change single factors within the complex setting of the intact organism. However, the potential influence of variations in the genetic background is a well-known property even in these models.^7,8^ In a creative approach, Marchal and colleagues turned this limitation into a tool to explore the effect of genetic background on a well-described pathogenic genetic variant found in patients with Brugada syndrome. The authors employed two different mouse strains with the murine equivalent of the human SCN5A-1795insD mutation. In previous studies, the same group has shown that mice carrying this mutation recapitulate key electrophysiological characteristics of Brugada syndrome.^9^ In the current publication, differences observed between the experimental groups in terms of electrophysiological and structural abnormalities illustrate that genetic background has a modulatory effect on the phenotype resulting from the Scn5a-1798inD mutation. Furthermore, young and old mice were compared, with one strain showing clear age-dependent development of structural changes.

Besides paving the way for further work to understand the mechanisms underlying the observed effects of age and genotype, this publication provides valuable reminders for both clinicians and basic scientists. The results add important information to the debate on the genotype–phenotype ‘mismatch’ and genetic modifiers in the Brugada syndrome that have been observed even in patient-specific cardiomyocytes derived from human-induced pluripotent stem cells.^10^ Although still categorized as a monogenic channelopathy, it has become increasingly clear that other genes and SNPs crucially influence the phenotype expression.^11,12^ This is perhaps most clearly illustrated by the development of polygenic risk scores, which currently seems to move the field in a promising direction after years of disputing candidate genes associated with the disease.^13^ Studies like the current one could move the field towards a deeper understanding of the underlying genetic basis for differences in phenotype.

Explorations of differences in phenotype despite the same underlying mutation are especially interesting for Scn5a-1798inD mutation, as the authors of the current article have previously shown that non-genetic factors, such as co-morbidities, might also have a modulatory effect.^14^ The authors should again be complimented for using a translational approach to pursue their earlier findings to gain a deeper understanding of this aspect of the disease. The previous results become even more interesting as the current study shows an age-dependent phenotype expression that was different between the two mice strains. As co-morbidities of course increase with age, progression in some patients with the underlying genotype might be expected. It is further worth noting that the authors even show differences between male and female mice, with less fibrosis in the females. Together, the results from the current article point to a complex interaction between age, genotype, and external factors to explain the phenotypic expression of this mutation. Whether the same applies to other genetic variants associated with the Brugada syndrome could be explored in a similar approach.

The use of animal *in vivo* models is essential for the transfer of new knowledge to clinical practice.^15^ However, the translation of insight from mouse to human should indeed be made with great caution. Nevertheless, an interesting finding in the current article is that structural and electrophysiological aspects of the pathology associated with the Brugada syndrome are not necessarily depending on the same factors. It is tempting to speculate that this might reconcile seemingly opposing views about the Brugada syndrome and explanations for its variations in phenotypic expression. As is well known to the readers of *Europace*, one theory explained the typical Brugada ECG pattern by transmural repolarization differences,^16^ whereas others focused on depolarization and conduction deficits,^17^ or even on discontinuous conduction throughout the myocardium.^18^ Importantly, the individual hypotheses are not mutually exclusive, as different factors can contribute to the phenotype in various patients. The current paper indicates that differences in genotype and exposure to external factors might promote different aspects of the pathophysiology, and thereby induce different abnormalities with a final common electrophysiological expression. This discussion has centred around the physiology of the right ventricle and especially the right ventricular outflow tract,^19^ which also illustrates an obvious limitation of mouse models where detailed studies of regional anatomy and physiology is difficult. Such studies will need to be pursued in larger mammals and humans for further mechanistic insight.

The current study also provides an important reminder to scientists employing mouse models for translational research. The background strain and measures to avoid genotypic drift in these inbred models can no longer be overlooked when reporting results. As a minimum, the specific background should be described sufficiently for other researchers to compare with their model, and descriptions of routines in the reporting lab to avoid untoward genetic drift should become a requirement. To our knowledge, clear rules in the form of guidelines are not available even though previous papers have emphasized the role of genetic background in the final phenotype and, thus, in the suitability of the selected model for concrete purposes.^20^

The current study should also be an inspiration for research not to simply resign when models unexpectedly ‘loose’ their phenotype over time, but instead use it as an opportunity to identify new factors with importance for the phenotype expression. Rightly heralded as a key tool for the reductionist approach in mechanistic studies employing animal models, transgenic mouse models also illustrate the complexity of biology and the difficulties in truly manipulating only one factor without affecting others at the same time. This lesson should stand as a humbling reminder to the clinician facing patients with varying phenotypes, as well as the translational scientist employing mouse models to elucidate biology.
